# Exploring the Functional Properties of Sodium Phytate Doped Polyaniline Nanofibers Modified FTO Electrodes for High-Performance Binder Free Symmetric Supercapacitors

**DOI:** 10.3390/polym13142329

**Published:** 2021-07-15

**Authors:** Sami Ur Rahman, Philipp Röse, Anwar ul Haq Ali Shah, Ulrike Krewer, Salma Bilal, Shehna Farooq

**Affiliations:** 1 National Centre of Excellence in Physical Chemistry 1, University of Peshawar, Peshawar 25120, Pakistan; samiurrahman720@gmail.com (S.U.R.); shehna.farooq@uow.edu.pk (S.F.); 2 Karlsruhe Institute of Technology (KIT), Institute for Applied Materials—Electrochemical Technologies (IAM-ET), 76131 Karlsruhe, Germany; ulrike.krewer@kit.edu; 3 Institute of Chemical Science, University of Peshawar, Peshawar 25120, Pakistan; anwarulhaqalishah@uop.edu.pk; 4 Department of Chemistry, University of Wah, Punjab 47040, Pakistan

**Keywords:** polyaniline, FTO-composite, nanostructure, supercapacitor, conductivity, electrochemical study

## Abstract

The performance of high-rate supercapacitors requires fine morphological and electrical properties of the electrode. Polyaniline (PANI), as one of the most promising materials for energy storage, shows different behaviour on different substrates. The present study reports on the surface modification of fluorine doped tin oxide (FTO) with the sodium phytate doped PANI without any binder and its utilization as a novel current collector in symmetric supercapacitor devices. The electrochemical behaviour of the sodium phytate doped PANI thin film with and without a binder on fluorine doped tin oxide (FTO) as current collector was investigated by cyclic voltammetry (CV). The electrode without a binder showed higher electrocatalytic efficiency. A symmetrical cell configuration was therefore constructed with the binder-free electrodes. The device showed excellent electrochemical performance with high specific capacities of 550 Fg^−1^ at 1 Ag^−1^ and 355 Fg^−1^ at 40 Ag^−1^ calculated from galvanostatic discharge curves. The low charge transfer and solution resistances (R_CT_ and R_S_) of 7.86 Ωcm² and 3.58 × 10^−1^ Ωcm², respectively, and superior rate capability of 66.9% over a wide current density range of 1 Ag^−1^ to 40 Ag^−1^ and excellent cycling stability with 90% of the original capacity over 1000 charge/discharge cycles at 40 Ag^−1^, indicated it to be an efficient energy storage device. Moreover, the gravimetric energy and power density of the supercapacitor was remarkably high, providing 73.8 Whkg^−1^ at 500 Wkg^−1^, respectively. The gravimetric energy density remained stable as the power density increased. It even reached up to 49.4 Whkg^−1^ at a power density of up to 20 Wkg^−1^.

## 1. Introduction

The steadily increasing consumption of fossil fuels and their rising prices have raised concerns about the rapid depletion of existing fossil fuel reserves and the associated alarming greenhouse gas emissions and environmental pollution. It is therefore important to develop environmentally friendly energy generation and storage devices [1] that can store and release large amounts of energy and have a long lifetime [2,3]. Currently, conventional batteries are the dominant energy sources due to their sufficient energy density, but still have shortcomings in terms of power density and a short lifetime [4]. Supercapacitors are promising energy storage devices that offer a solution to achieve high power density, fast charging and discharging, and an extended lifetime [5]. These properties have led to supercapacitors being used as the dominant power source in portable electronic devices, medical supplies, memory backup systems, and for higher power requirements such as electric vehicles [6]. The current challenges in developing supercapacitors are to further increase their energy density while maintaining their ability to achieve high power density and long life. The electrode material is the critical component that determines the performance of the supercapacitor. Therefore, extensive research has focused on the design and fabrication of electrode materials that have shortened ion diffusion paths, enabling high performance, and large capacitance [7,8].

Currently, three types of materials are commonly used to make electrodes in supercapacitors—carbon-based materials, transition metals, and conductive polymers [9]. For carbon-based materials such as activated carbon, carbon nanotubes, and reduced graphene oxide, the energy storage depends on the electrostatically stored charge between the interfaces between the electrolyte and the electrode. However, some factors, including low specific capacitance due to higher interfacial resistance and inherent resistance of the binder, affect the carbon-based materials that could limit the performance of the device to be manufactured [10]. Therefore, research is focused on electrodes based on transition metal oxides for supercapacitors such as RuO_2_, MnO_2_, NiO, VO, Co_3_O_4_, etc. [11]. The storage of energy in transition metal oxide electrodes depends on fast and reversible redox reactions at the surface of the active materials. Nevertheless, these transition metal oxides have high electrical resistivity, which in turn affects the performance, resulting in moderate specific capacitance and low power density [12].

The third type of electrode materials in supercapacitors are intrinsically conductive polymers. The energy storage mechanism of these polymers is based on a process known as doping [13]. These conductive polymers can improve their energy storage capacity and reduce self-discharge due to their high doping and de-doping rates during the charge–discharge process [14]. Conductive polymers of pseudocapacitive materials have the advantages of high electrical conductivity and very low cost compared to transition metal oxides [15].

PANI has been used either as a conductive agent or directly as an electrode material in energy storage technologies due to its tuneable pseudocapacitive performance based on its different oxidation states. Furthermore, with its properties of high conductivity, good redox reversibility, environmental stability, and different synthesis routes, PANI offers the potential for further practical applications [16]. 

Generally, PANI is synthesised from aniline monomer either by chemical oxidative polymerisation or direct electrochemical oxidative polymerisation in the presence of a dopant and an oxidising agent [16,17]. PANI produced by chemical oxidative polymerisation often requires binders to be mixed with the synthesised polymer to produce an electrode from the powder polymer material [18]. The use of binders leads to an increase in electrical resistance and thus to a decrease in the specific capacity of the produced electrodes [18]. In addition, the current collector plays an important role as a substrate for the active materials in supercapacitors to effectively collect and transport electrons to and from the electroactive materials during the charging and discharging process [19].

It has been observed that the different PANI materials act differently on different current collectors. Deshmukh et al. [20] reported the capacitive behaviour of a PANI thin film deposited on a fluorine-doped tin oxide (FTO) substrate by microwave-assisted chemical means. The specific capacitance of the PANI thin film electrode was 546 Fg^−1^ at a scan rate of 5 mVs^−1^ and 50 Fg^−1^ at 100 mVs^−1^. Yogesh et al. [21] coated 3D PANI on Toray paper (current collector) as a substrate and produced a device with a specific capacitance of 350 Fg^−1^ at 40 Ag^−1^. Razali et al. [14] electrochemically deposited PANI nanorods on electro-etched carbon cloth and reported a specific capacitance of 357 Fg^−1^ at 0.5 Ag^−1^ and 323 Fg^−1^ at 10 Ag^−1^. PANI-DBSA synthesised by interfacial polymerisation was used to fabricate a symmetrical supercapacitor device with gold sheet as current collector and showed a specific capacitance of 412 Fg^−1^ and 215 Fg^−1^ at 1 and 10 Ag^−1^, respectively [22]. Additionally, an aqueous supercapacitor made with PANI-GO nanocomposites on gold sheets showed a specific capacitance of the device of 264 Fg^−1^ and 222 Fg^−1^ at 1 Ag^−1^ and 10 Ag^−1^, respectively [23]. 

The difference in the capacitance of different PANI materials on different substrates is obvious, as it depends on both the material morphology and the substrate used as a current collector [24]. Therefore, both the electroactive materials and the current collectors have been scaled down to the nanoscale to provide unique properties leading to high performance nanostructured electrodes for pseudocapacitive material [25]. However, an analysis of the same PANI material on different substrate is still missing.

Recently we have reported a very simple, fast and green synthesis route for polyaniline nanofibers doped with sodium phytate [26]. Not only the method was extremely promising, but the resulting PANI nanofiber showed good relationship between their structure and properties with excellent energy storage characteristics on the surface of gold sheets.

FTOs are commonly used substrates for supercapacitors electrodes. In this regard, the present work is based on the fabrication of binder-free symmetrical supercapacitor devices using nanostructured sodium phytate doped PANI as the active material and FTO as the current collector. The fabrication of PANI-functionalised electrodes typically requires the addition of binder and a suitable solvent to the polymer powder to form a stabilised and homogeneous coating solution. However, the weak interaction and interface problem between the binder and the active material in addition to the low ion/electron conductivity results in the reduction of specific capacitance. Sodium phytate fulfils two functions; it acts as a dopant and as a crosslinker. Its use leads to the formation of a nanoscale PANI fibre network with a large surface area, which enables efficient charge storage. At the same time, the fibres allow easy bonding to the FTO without the need for a binder. Two identical FTO glasses were used as current collectors onto which active material was dropped. The device was subjected to electrochemical characterisations. The device showed excellent specific capacitance, capacitance retention, stability, energy density and power density with very low charge transfer resistance (R_ct_) and solution resistance (R_s_).

## 2. Materials and Methods

### 2.1. Materials and Synthesis

Sodium phytate-doped PANI was synthesised from commercially available materials: Aniline (C_6_H_5_NH_2_), sodium phytate (C_6_H_17_NaO_24_P_6_), ammonium persulphate ((NH_4_)_2_S_2_O_8_, and dimethylformamide (C_3_H_7_NO) were purchased from Sigma Aldrich (St. Louis, MI, USA) and sulphuric acid (H_2_SO_4_) was provided by Scharlu (Barcelona, CAT, Spain). Aniline was freshly distilled twice to remove all kinds of impurities. After distillation, the aniline was stored in a refrigerator for further use. The other chemicals were used as received. Deionised water was used for sample synthesis and washing. Fluorine-doped tin oxide (FTO) glass (13 Ω/sq) was obtained from Solaronix (Aubonne, Switzerland).

### 2.2. Synthesis of Sodium Phytate Doped PANI

Sodium phytate doped PANI was synthesized according to our recently reported procedure [26]. Sodium phytate solution 5% (*w*/*v*) was prepared in H_2_O at room temperature (solution A). Then, 2.5 mL from the respective sodium phytate solution was mixed with 5.50 mmol aniline in 5 mL H_2_O. A second solution containing ammonium persulfate (1.0 mM in H_2_O) was prepared (solution B). These solutions were kept in refrigerator for 15 min. Then, polymerization was performed by adding 1.0 mL from solution A to 0.5 mL of solution B in a reaction vessel followed by fast and thorough mixing. After 5 min, the colour of the mixture turned dark green indicating the formation of PANI. The mixture was filtered, washed with acetone, and was dried under vacuum for further characterization and fabrication of the symmetric supercapacitor device.

### 2.3. Fabrication of Working Electrode

PANI based working electrode was prepared by dispersing 10 mg of PANI-samples in 5 mL DMF and stirred thoroughly at room temperature then placed in an ultrasonic sound bath for 30 min. The respective FTOs were coated with PANI-DMF suspension. Then, the solvent was led to evaporate slowly at 25 °C for 12 h resulting in a thin and even layer of dry PANI on the FTOs surface. The coated PANI-FTOs electrode was dried under vacuum at 80 °C for 24 h to remove residual solvent. After drying, the active PANI material on the FTO electrode surface was 2 mg cm^−2^ for each analysis. The same procedure was used to prepare the binder-supported FTO-PANI electrode by dispersing 9 mg PANI in 5 mL DMF. Then, 1 mg of binder was added to the dispersion with vigorous stirring.

### 2.4. Fabrication of FTO-PANI Supercapacitor Device

**Figure 1:**
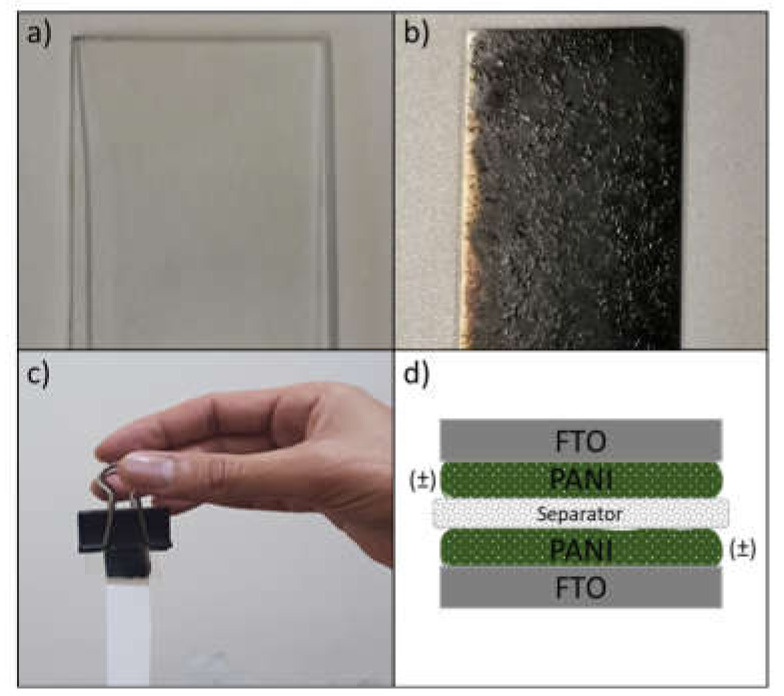
(**a**) Uncoated FTO (2 × 2 cm^2^), (**b**) PANI-coated FTO, (**c**) fabricated device, and (**d**) schematic illustration of device.

A separator (Whatmann 1441-047, pore size 20 μm) was placed between two prefabricated FTO electrodes in such a way that one end protruded to provide the electrolyte, constantly. This sandwich-like cell was then fixated using plastic clips (Figure 1c). The complete construction is as follows: FTO/PANI-separator-PANI/FTO (Figure 1d).

### 2.5. Characterization

#### 2.5.1. Structural and Morphological Characterization

Characterisation of the surface morphology, elemental composition, and mapping of the synthesised PANI was carried out using Helios G4 CX FEI Deutschland GmbH (Berlin, Germany). Nitrogen adsorption-desorption isotherms were measured using the Brunauer–Emmett–Teller (BET) method on a Quanta Chrome Instrument surface area analyser (Version 11.04, Boynton Beach, FL, USA). The pore size distribution was determined from the adsorption branches using the Barret–Joyner–Halenda (BJH) method. X-ray diffraction (XRD) spectrum was recorded by using Cu Kα radiations (λ = 1.5405 A°) on X-ray diffractometer (JEOL, Tokyo, Japan) with a scan rate of 0.05^0^/s. Conductivity was determined in the form of pellets using a four-probe conductometer (Jandel RM 3000, Jandel Engineering Ltd., Linslade, Beds., UK) equipped with a potentiostat. The pellets (diameter: 13 mm and thickness: 5 mm) were produced using a hydraulic press with a pressure of 15 tonnes.

#### 2.5.2. Electrochemical Characterizations

Electrochemical measurements including cyclic voltammetry (CV), galvanostatic discharge (GCD), and electrochemical impedance spectroscopy (EIS) were performed in 1 M H_2_SO_4_ using ZRA/Potentiostat/Galvanostat Reference 3000 (Gamry Instruments, Warminster, PA, USA). A three-electrode system was used for cyclic voltammetry. The potential was referred to a Ag/AgCl (sat. KCl_aq_) electrode, while gold coil was used as counter electrode. The potential window ranged between −0.2 V and 0.8 V. Specific capacities were calculated from the CV curves using Equation (1) [27]:

(1) $$C_{sp}=\frac{I}{mv}$$

where *I* signify the current, *m* depicts the mass of the active material, and *v* represents the scan rate. The loading of active material is 2 mg cm^−2^ (dry weight).

The capacitive performance of the fabricated device by GCD was tested in a two-electrode system in which both ends of the FTO electrodes were clamped using clips connected to the device. During the measurement, the cell was suspended upside down in a beaker containing 1 M H_2_SO_4_ electrolyte solution, with the protruding filter paper immersed in the electrolyte to provide electrolyte continuously by capillary action. GCD measurements were performed between −0.2 V to 0.8 V at different current densities. The specific capacitances, energy densities and power densities were calculated using Equations (2)–(4) [28].

(2) $$C_{sp}=2\times \frac{I\times \Delta t}{\Delta V\times m}$$

Where, factor 2 is due to the symmetric cell setup. Δt is the duration of the discharge process in seconds (s), *I* refers to current (A), ΔV indicates the potential window in volts (V), and *m* denotes the mass of the electroactive material in grams. The gravimetric energy density is described as follows:

(3) $$E\left(\frac{Wh}{kg}\right)=\frac{1}{2}C_{sp}\Delta V^{2}\times \frac{1000}{3600}$$

where *E* is the gravimetric energy density with unit of Wh kg^−1^, *C_sp_* designates specific capacitance (Fg^−1^) obtained from Equation (1) or (2), and ΔV tells the potential window in volts (V). From the gravimetric energy density, the gravimetric power density P can be calculated with Equation (4):

(4) $$P\left(\frac{W}{kg}\right)=\frac{E}{\Delta t}=\frac{I\Delta V}{2m}\times 1000$$

The gravimetric power density contains the unit W kg^−1^, *E* is energy density obtained from Equation (3), Δt is the period of discharging process in seconds (s), *I* refer to current (A), ΔV tells the potential window in volts (V), and *m* denotes mass of the electroactive material in grams. 

Electrochemical Impedance Spectroscopy (EIS) was also used to characterize the symmetric supercapacitor device for charge transfer resistance (*R_ct_*) and solution resistance (*R_s_*). The frequency ranged from 100 mHz to 1 MHz using a perturbation amplitude of 5 mV_rms_ and a DC-potential of 0.5 V.

## 3. Results and Discussion

### 3.1. Structural and Morphological Characterization

**Figure 2:**
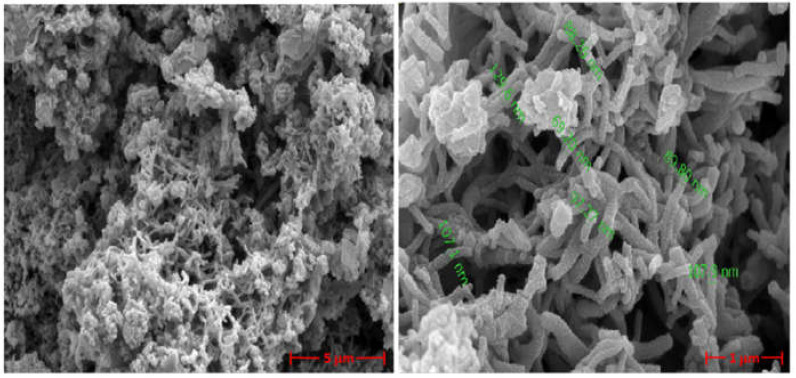
SEM images of the surface of sodium phytate doped PANI at a magnification of 20 k (**left**) and 80 k (**right**).

Figure 2 shows the SEM images of the synthesised PANI doped with sodium phytate at 20 k and 80 k magnification. It is a long, uniform, interconnected fibre structure with a rough and porous morphology without the presence of lateral fibres with twists, random aggregates, and breakage of the fibres ranging from 69 nm to 129 nm in diameter.

The morphology of the synthesised PANI is directly related to the surface area, the range of pore size distribution and the volume of the pores, as reported in our previous study [26]. The PANI sample synthesised with optimised parameters showed a significantly higher surface area (230.5 m^2^g^−1^), pore size distribution in the range of 15.0–19.5 nm, and pore volume of 0.046 cm^3^g^−1^. These results suggest that such a porous and cross-linked PANI nanofiber structure may be more advantageous than wires and particles for electrical and capacitive properties when used as electrode material for supercapacitors (see Section 3.2). This is due to the large open channels of the pores with rough surface within the structures [29], as the fibrous and rough property of PANI provides a large surface area favourable for the transport of electrons and ions and is beneficial for good electrocatalytic properties [30,31,32].

**Figure 3:**
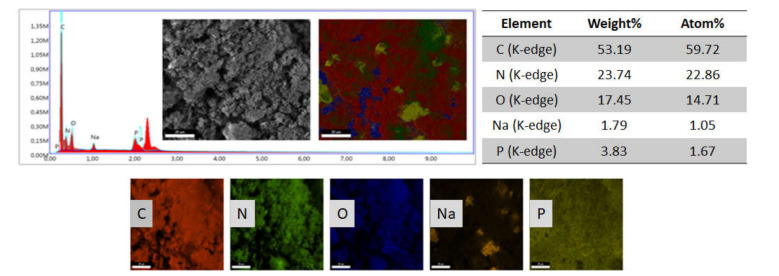
EDX spectrum of PANI nanofibers.

To investigate the elemental composition of PANI doped with sodium phytate, Energy Dispersive X-ray (EDX) analysis was used (Figure 3). The EDX results show that PANI contains C, O, Na, N, and P. The detection of Na and P is evidence of the successful incorporation of the sodium phytate dopant into the polymer backbone. The presence of Na and P in doped PANI minimises the barrier height, and conjugation in the polymer chain creates a deep interaction for charge delocalisation in and between different chains. This is believed to improve the electrical conductivity of the doped PANI [33]. The homogeneous distribution of C, N, O, Na, and P is also determined by the EDX elemental map.

**Figure 4:**
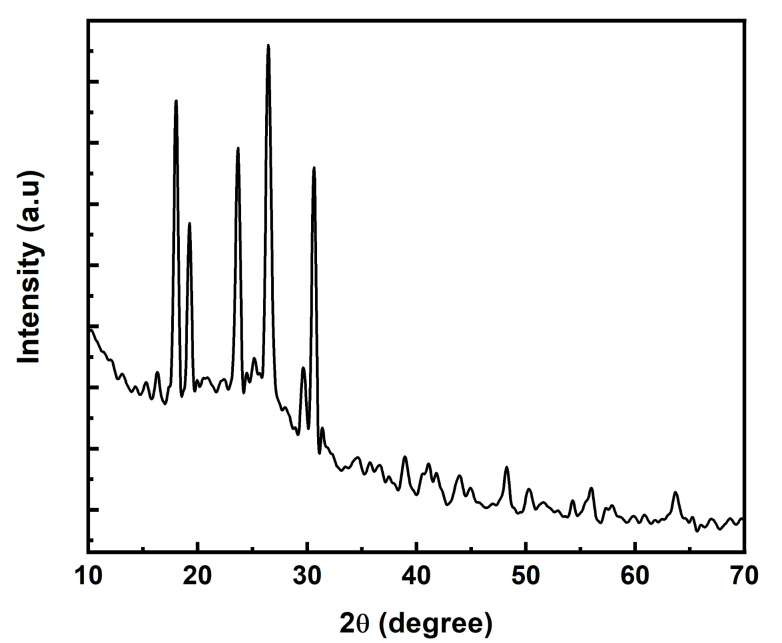
XRD spectrum of sodium phytate doped PANI.

XRD examination was done to evaluate the molecular order with regard to crystallinity, as charge transport in CPs enhance by increasing molecular order. X-ray scattering pattern of sodium phytate doped PANI presented in Figure 4. It can be noted that XRD spectra shows intense peaks at 2θ = 17°, 20.5°, 23°, 26°, 29.6°, and 30°. The peaks at 2θ = 17°, 23°, and 29.6° are characteristics of emeraldine salt state of PANI [34]. The peaks at 2θ = 20° and 2θ = 26° corresponds to the parallel and vertical periodic intervals of PANI chains/backbone, respectively. The diffraction peaks observed at 26°, 29.6°, and 30° reflect the aniline and dopant interaction during the polymerization and suggested incorporation of dopant into polymer backbone [34]. From the XRD diffractogram of sodium phytate doped PANI, it can be seen that all the peaks are very intense. These intense peaks count for the best structural ordering and hence may exhibit higher crystallinity in the backbone of the polymer [35]. These properties are responsible for intermolecular transport of the ionic species alongside the polymer chain and a little intermolecular hopping owing to close and better packing of the material. Consequently, this high crystallinity of the material leads to high conductivity and high electrocatalytic activity [36,37]. 

Literature study reveals that by using commercial phytic acid solution as a dopant, the similar behaviour of XRD pattern is obtained with low crystallinity as for other reported organic and inorganic acids doped PANI. Xiaohui Gao and his co-workers [33] investigated comparison of undoped PANI, H_3_PO_4_ doped PANI, and commercial phytic acid solution doped PANI. They reported same XRD spectra with low crystallinity for doped PANI compared to undoped PANI. Santos et al. [38] also reported the similar behaviour of XRD spectra with low crystallinity for H_2_SO_4_ doped PANI and commercial phytic acid solution doped PANI. In the present study, behaviour of XRD pattern is different from those reported in the literature. Presence of high intense peaks reveals the high crystallinity of the prepared material. Therefore, our newly oxidative method along with sodium phytate as a novel dopant might be responsible for high crystallinity of PANI.

### 3.2. Electrochemical Characterization

#### 3.2.1. Electrical Conductivity

The electrical (DC) conductivity of a PANI pellet doped with sodium phytate was determined from the conductivity measurement multiplied by the geometric factor of the sample. The conductivity value at 25 °C of the synthesised PANI is 10 Scm^−1^. This remarkably high value could be due to good electron delocalisation in the conjugated polymer, which is prolonged by the formation of a nanoscale, fibrous, cross-linked network after using sodium phytate as a dopant. The fibre structure with a large surface area and pore volume as well as highly assimilated dopant content significantly increases the conductivity of PANI [26]. Due to this high conductivity value, PANI doped with sodium phytate is a potential candidate for use as an electroactive electrode material in electronic devices.

#### 3.2.2. Cyclic Voltammetry (CV)

**Figure 5:**
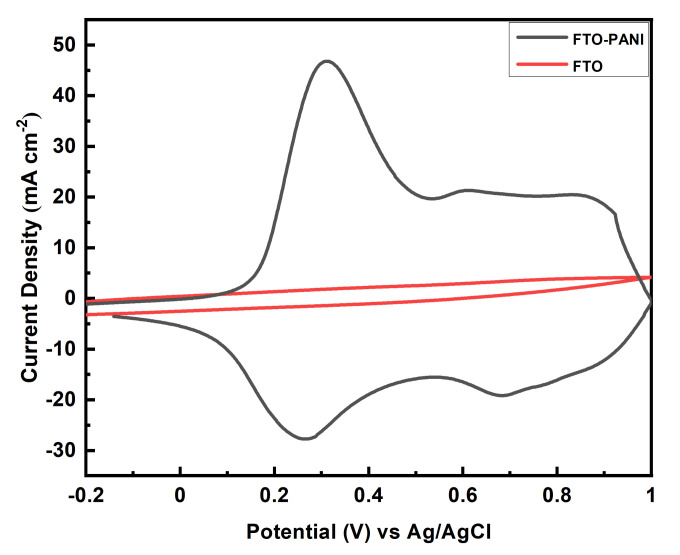
Cyclic voltammogram of FTO (black line) and binder free FTO-PANI (red line) in 1 M H_2_SO_2_ vs Ag/AgCl (KCl_sat_ in H_2_O) at 20 mVs^−1^.

Cyclic voltammetry (CV) measurements were performed to investigate the electrochemical efficiency and capacitive behaviour of the FTO-PANI. Figure 5, shows the voltammogram of FTO and an FTO-PANI coated electrode at a scan rate of 20 mVs^−1^. It can be clearly seen that there is almost no contribution of FTO to the capacitance of the cell. The PANI-coated electrode shows a quasi-rectangular CV curve with two sets of distinct redox activity as indicated by the two pairs of anodic and cathodic current peaks. This is the typical Faradaic energy storage mechanism of PANI, which can be found several times in the literature [39].

The first set of a redox couple which appears between 0 V and 0.3 V vs Ag/AgCl is associated with the conversion of the fully reduced leucoemeraldine base (LB) to the partially oxidized emeraldine salt (ES), and the second set of redox current peaks occurring between 0.6 V and 0.8 V vs Ag/AgCl pertains to the conversion of emeraldine salt (ES) to the fully oxidized pernigraniline form (PB). ES can be formed by the oxidation without the change in the number of hydrogen atoms attached to nitrogen atoms (the proton transfer is not involved). It occurs through the formation of a radical cation at *N*-position which is accompanied by the anions insertion to maintain the electroneutrality. On the other hand, protons are involved in the redox reaction associated with the second peaks. The depression in the CV curve around 0.5 V indicated the highest conducting state of the material. The shape of the CV curve showed a good capacitive nature of PANI, which consists of a combination of double layer capacitance and pseudocapacitance from the redox couples [40]. The combined high capacitance is related to the large surface area of the cross-linked nanofibrous and porous morphology. It indicates a fast ion transport rate and thus a high capacitance [41].

**Figure 6:**
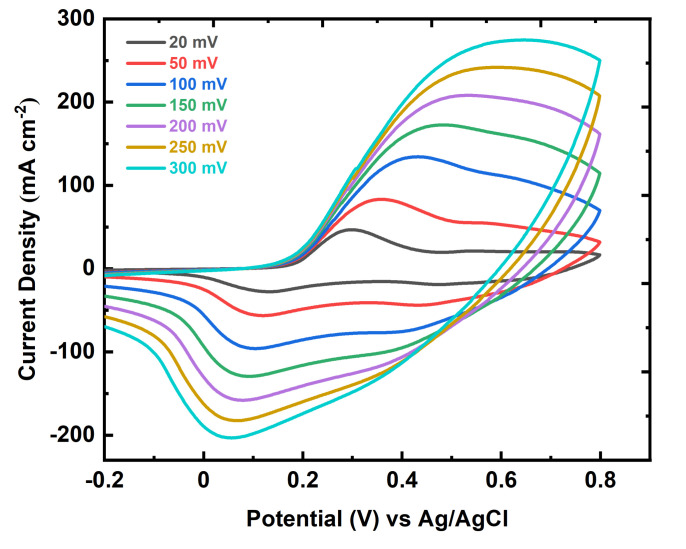
Cyclic voltammogram FTO-PANI in 1 M H_2_SO_4_ vs Ag/AgCl (KCl_sat_ in H_2_O) at various scan rates.

The capacitance behaviour of FTO-PANI was further investigated by recording the CV curves at a wide range of scan rates (20, 50, 100, 150, 200, 250, and 300 mVs^−1^). The cyclic voltammograms of binder free FTO-PANI electrodes are shown in Figure 6. The FTO-PANI thin film electrode (Figure 6) showed a good specific capacitance of 666.6 ± 2.1 Fg^−1^ at a scan rate of 20 mVs^−1^. The value of the specific capacitance decreased to 346.0 ± 2.5 Fg^−1^ when the scan rate was increased up to 300 mVs^−1^. The decrease in capacitance is attributed to the presence of internal active sites that cannot fully maintain redox transitions at higher scan rates.

This is probably due to diffusion limitation of the acidic electrolyte within the electrode fibre network, so that the parts of the surface electrode are not accessible at high discharge rates. It is therefore assumed that the specific capacitance obtained at the slowest sampling rate is closest to the full utilisation of the electrode material [42]. It is noteworthy that the electrode also shows excellent electrochemical behaviour over a wide range of scan rates. The CV curves of the electrode are quite stable and do not reflect any aberration even at such high sampling rates, indicating that PANI coated on FTO is very stable in terms of charge transfer [41,43].

**Figure 7:**
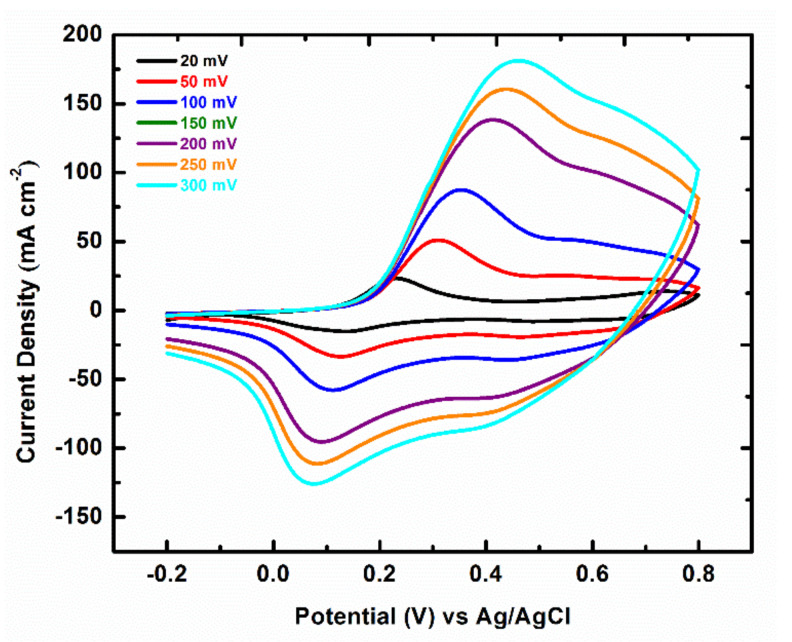
Cyclic voltammogram binder supported FTO-PANI in 1 M H_2_SO_4_ vs Ag/AgCl (KCl_sat_ in H_2_O) at various scan rates.

For comparison, the capacitance behaviour of the binder-supported FTO-PANI electrodes were also investigated by cyclic voltammetry over a wide range of scan rates analogous to the self-supported FTO-PANI electrodes (Figure 7) and the calculated specific capacitance values were compared with the binder-free electrode in Table 1. A similar trend for the sequence of capacitance values was observed for the binder-supported FTO-PANI electrode as for the unsupported FTO-PANI electrode. Thus, values of 344.0 ± 2.1 Fg^−1^ and 233.3 ± 2.5 Fg^−1^ were obtained for the specific capacitances at scan rates of 20 mVs^−1^ and 300 mVs^−1^, respectively. These values are lower than those for the self-supporting FTO-PANI electrodes. This could be due to the inclusion of the binder, which blocks part of the electrochemically active surface and also increases the resistance of the electrode, since it is insulating the electrode partially [19]. It is evident from the results that the electrochemical performance of the self-supporting FTO-PANI electrodes is significantly higher compared to the binder-supporting FTO-PANI electrodes.

#### 3.2.3. Fabrication of Symmetric Supercapacitor

**Figure 8:**
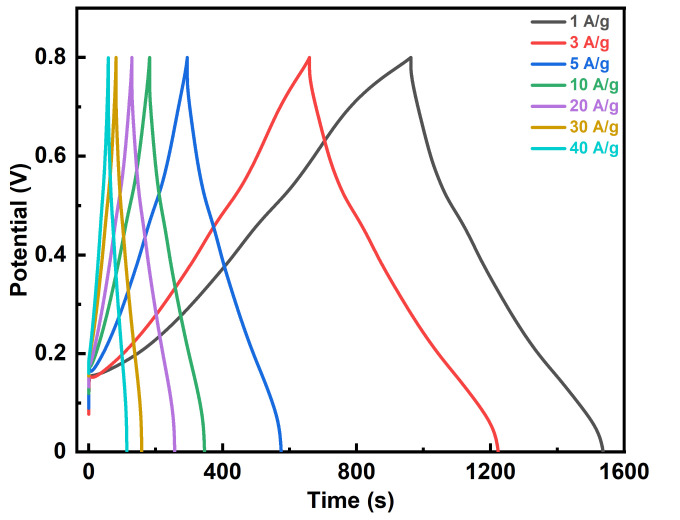
Galvanostatic charge–discharge curves of a symmetric FTO-PANI supercapacitor at 1, 3, 5, 10, 20, 30, and 40 Ag^−1^ in a three electrode setup.

A symmetrical cell setup with two identical binder-free FTO-PANI electrodes was used for charge–discharge studies. The device was tested at different current densities ranging from 1 Ag^−1^ to 40 Ag^−1^ (Figure 8).

The specific capacities for the different current densities were calculated from the discharge time curves (Table 2).

**Figure 9:**
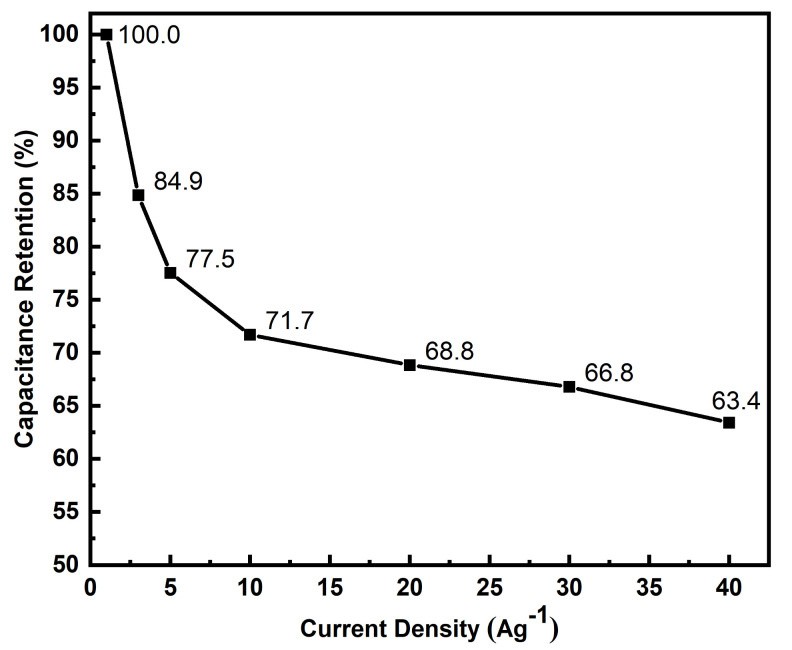
Respective specific capacitance retention values for a wide range of current densities ranging from 1 Ag^−1^ to 40 Ag^−1^.

Between 1 Ag^−1^ to 10 Ag^−1^, the specific capacitances decrease exponentially from 531.5 ± 2.2 Fg^−1^ to 397.5 ± 2.3 Fg^−1^, which corresponds to specific capacitance retention of 74% of the initial value. With progressively higher current densities, the decrease in specific capacitances became monotonically decreasing to 355.4 ± 2.2 Fg^−1^ at 40 Ag^−1^, indicating an overall loss of 176.2 Fg^−1^ (33%, Figure 9). Although the specific capacitance values vary over such a wide range of current densities, it is important to note that the material exhibits remarkably high specific capacitance and stable values especially at high current densities [14,20,21,22,23,41]. This is also evident when comparing the specific capacitances of various PANI and PANI-based symmetrical supercapacitors from the literature (Table 3).

**Figure 10:**
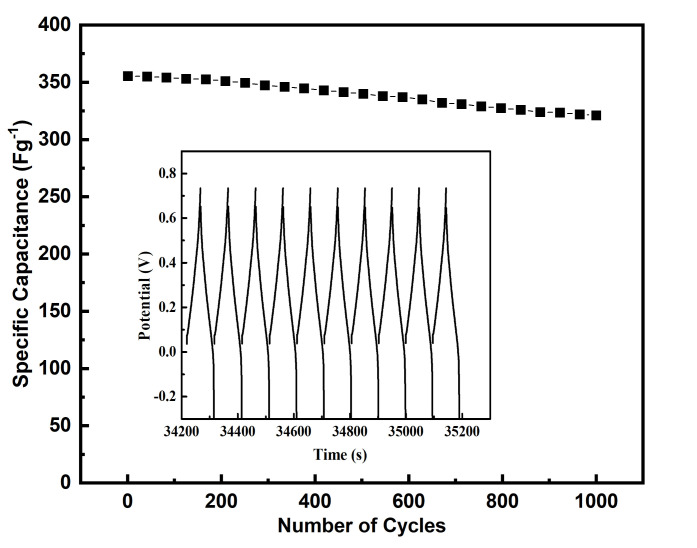
Cycle stability of an FTO-PANI based symmetric supercapacitor over 1000 galvanostatic charge–discharge cycles at 40 Ag^−1^. The inset shows the individual cycles progressing aging stage.

Cycling stability is a key factor in the operation of supercapacitors. Conductive polymers in supercapacitors often have limited cycling stability due to shrinkage and swelling. In this work, cycling stability was tested over 1000 cycles at 40 Ag^−1^. As can be seen in Figure 10, FTO-PANI shows excellent cycling stability (90%) without significant loss of specific capacitance at such a high current density. To our knowledge, 90% rate capability at 355.4 ± 2.2 Fg^−1^ at 40 Ag^−1^ has never been reported for PANI, especially for symmetrical cell setups.

**Figure 11:**
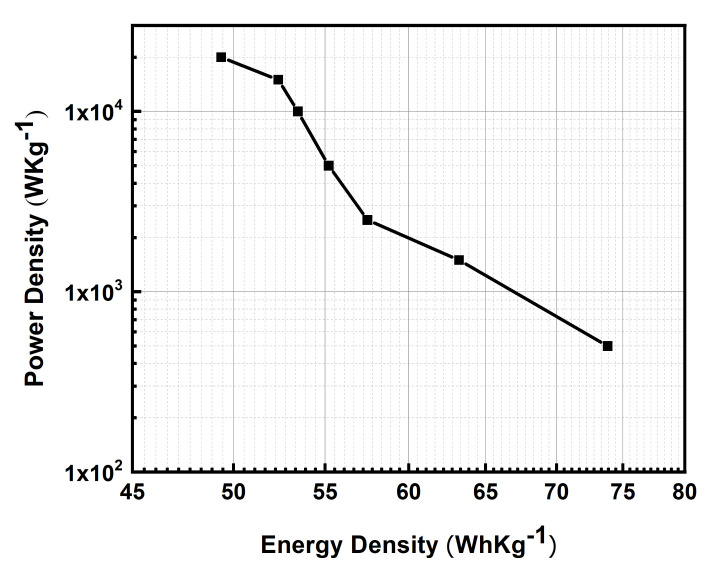
Ragone plot of the FTO-PANI supercapacitor for current densities between 1 Ag^−1^ (top left side) to 40 Ag^−1^ (bottom right side).

For further performance evaluation of the FTO-PANI supercapacitor, a Ragone projection was used and the relationship between gravimetric energy and power density was examined (Figure 11) [49]. The gravimetric energy density and power density were calculated using Equations (3) and (4) [28]. The plot shows the energy and power density of the device at the measured current densities. It can be seen from the plot that there is a small loss of energy density when the power density is greatly increased. The device delivered a gravimetric energy density of 73.82 Whkg^−1^ at a power density of 500 Wkg^−1^. More importantly, the energy density was quite stable with the increase in power density. The energy density reached up to 49.35 Whkg^−1^ even at a power density of 20 kWkg^−1^, which is much higher than most current commercial supercapacitors [28] and in previous reports based on a PANI-based system [14,20,21,22,23]. This makes this FTO-PANI device very interesting for applications with high power density requirements.

It is noteworthy that the factors of morphology and electrical conductivity play such a significant role in improving the specific capacitance and charge storage capability of the materials [28].

#### 3.2.4. Electrochemical Impedance Spectroscopy (EIS)

**Figure 12:**
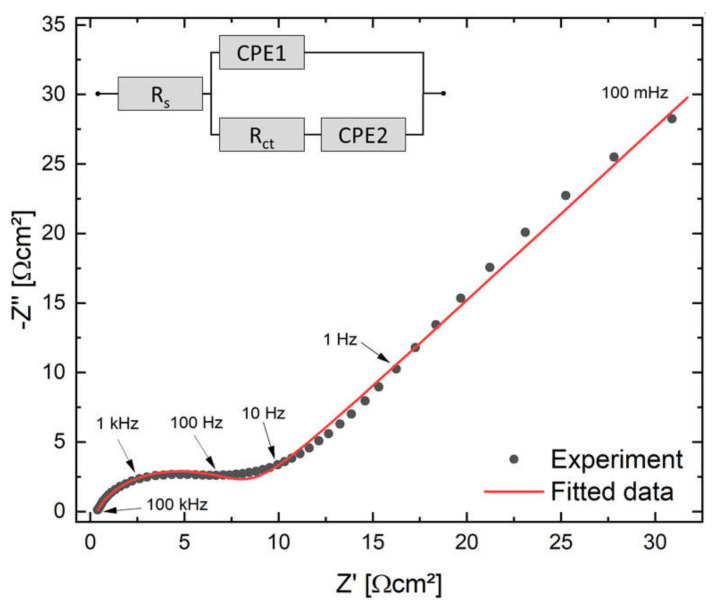
Nyquist plot of the FTO-PANI supercapacitor at *E*_DC_ = 0.5 V. The inset displays the equivalent circuit for the device.

To evaluate the charge transfer process at the electrode/electrolyte interface, the symmetrical FTO-PANI supercapacitor was analysed by EIS at open-circuit potential in the frequency range 100 mHz to 1 MHz. The corresponding Nyquist diagram is shown in Figure 12.

The recorded EIS spectrum can be divided into three sections: (1) the x-axis section contains the series resistance from FTO substrate, wiring, electrical and ionic resistance through electrode and electrolyte (~100 kHz), (2) an open semicircle in the high frequency range (100 kHz to 10 Hz), which is often associated with the double-layer and Faradaic charge transfer processes of PANI, and (3) a monotonically increasing slope at frequencies below 10 Hz, which is typically associated with ion transport limitations and other slow diffusion and absorption processes [40]. The steeper the sloping line, the more ideal the capacitive behaviour is given [34,50].

In order to determine the most important parameters, the experimental data were fitted with an equivalent circuit model containing the series and charge transfer resistance *R_S_* and *R_CT_* and two constant phase elements CPE1 and CPE2, which can be attributed to the double layer capacitance at the electrode-electrolyte interphase and the pseudo-capacitance of the PANI [40]. The FTO-PANI supercapacitor exhibited a very small series resistance of 358 mΩcm^2^ and a charge transfer resistance of 7.86 Ωcm^2^. These values indicate that the cell possesses a very high conductivity and electron transfer rate between the different cell components. The result agrees with those from the electrical conductivity measurements. Furthermore, the good pseudocapacitive behaviour of the supercapacitor was reflected in the values of the CPE elements. They were 26.1 mS and 278 μS for CPE1 and CPE2, respectively, and a value of 0.57 and 0.78 were found for the exponents n. The values show that the pseudo-capacitance of the conducting PANI is the main source of energy storage, while conventional capacitive effects play only a minor role.

## 4. Conclusions

PANI fibres doped with sodium phytate can be used as effective materials for binder-free surface modification of various substrates, including FTO, resulting in remarkable pseudocapacitive properties that make it a promising material for energy storage. A binder is often needed to attach the polymer to the FTO surface and increase the stability of the electrode. We were able to show that this is not necessary for sodium-doped PANI nanofibres. The omission of the binder led to an increased electrochemical activity. Thus, the specific capacitance of the PANI-FTO electrode without binder, calculated from cyclic voltammetry measurements in a three-electrode setup, was 666.6 ± 2.1 Fg^−1^ at 20 mVs^−1^ and 346.0 ± 2.5 Fg^−1^ at 300 mVs^−1^, respectively, indicating very good rate capability. The values obtained agreed with the observations in electrochemical impedance spectroscopy and showed very low values for series and charge transfer resistance as well as good pseudocapacitive properties. The use of binder in the same cell setup, on the other hand, resulted in a reduction of the specific capacitance between 34% and 49%, depending on the scan rate.

With regard to real applications, the analysis of the binder-free FTO-PANI composite for the construction of symmetrical supercapacitors showed that this combination has the potential for practical applications. In the case of the symmetrical device, the specific capacitances were 531.5 ± 2.2 Fg^−1^ at a current density of 1 Ag^−1^ and 355.35 ± 2.195 Fg^−1^ at 40 Ag^−1^, respectively. The sample showed excellent capacitance retention of 90% over 1000 cycles at 40 Ag^−1^. In addition, the FTO-PANI supercapacitor showed a high energy density of 73.8 Whkg^−1^ at a power density of 500 Wkg^−1^ and was held at 49.4 Whkg^−1^ at a power density of 20 kWkg^−1^.

## Figures and Tables

**Table 1:** Specific capacitances (C_sp_) values of binder free and binder supported FTO-PANI electrode with binder at wide range scan rates.

Scan Rate/ mVs^−1^	Specific Capacitance (Fg^−1^) of FTO-PANI Electrode without Binder	Specific Capacitance ^a^ (Fg^−1^) of FTO-PANI Electrode with Binder
20	666.6 ± 2.1	340.0 ± 2.1
50	617.6 ± 2.2	331.6 ± 2.3
100	548.7 ± 2.1	302.3 ± 2.6
150	481.7 ± 2.3	279.0 ± 2.4
200	428.6 ± 2.3	261.2 ± 2.5
250	384.0 ± 2.5	246.4 ± 2.3
300	346.0 ± 2.5	228.3 ± 2.4

^a^ Specific Capacitances were determined by integration of the cyclic voltammograms.

**Table 2:** Specific capacitances, retentions and respective gravimetric energy and power densities of FTO-PANI symmetric supercapacitor at varying current densities.

Current Density/ Ag^−1^	Specific Capacitance ^a^ /Fg^−1^	Capacitance Retention ^b^	Energy Density/Whkg^−1^	Power Density/ Wkg^−1^
1	531.5 ± 2.2	-	73.8	500
3	455.3 ± 2.1	86%	63.2	1500
5	413.8 ± 2.0	78%	57.5	2500
10	397.5 ± 2.3	75%	55.2	5000
20	385.0 ± 2.1	72%	53.5	10,000
30	377.2 ± 2.0	71%	52.4	15,000
40	355.4 ± 2.2	69%	49.4	20,000

^a^ Specific Capacitances were determined from the discharge slopes of the respective galvanostatic charge–discharge-measurements; ^b^ The retention values are relative to the measurement with the lowest current density (1 Ag^−1^).

**Table 3:** Comparison of the specific capacitance of the symmetrical FTO-PANI supercapacitor with reported supercapacitors electrodes from the literature.

Materials ^a^	Current Density	Specific Capacitance	Year	Reference
PANI-DBSA-Gold	10 Ag^−1^	215 Fg^−1^	2019	[22]
PANI-GO-Gold	1 Ag^−1^	264 Fg^−1^	2020	[23]
FTO/PANI	0.1 Ag^−1^	176.29 Fg^−1^	2018	[44]
PANI-NR coated-FTO	1 Ag^−1^	106 Fg^−1^	2020	[45]
PANI-FTO//PANI-TiO-FTO	5 Ag^−1^	419 Fg^−1^	2019	[46]
FTO/PANI	0.2 Ag^−1^	155.65 Fg^−1^	2021	[47]
PANI-ACP	1 Ag^−1^	402 Fg^−1^	2020	[48]
PANI-FTO	1 Ag^−1^	531.5 ± 2.2 Fg^−1^	present work	
	40 Ag^−1^	355.4 ± 2.2 Fg^−1^		

^a^ In the cell setups 1 M H_2_SO_4_ was used as electrolyte.

## Data Availability

The data presented in this study are available on request from the corresponding author.
